# Mechanical plasticity of cell membranes enhances epithelial wound closure

**DOI:** 10.1103/physrevresearch.6.l012036

**Published:** 2024-02-22

**Authors:** Andrew T. Ton, Arthur K. MacKeith, Mark D. Shattuck, Corey S. O’Hern

**Affiliations:** 1 Department of Physics, Yale University, New Haven, Connecticut 06520, USA; 2 Integrated Graduate Program in Physical and Engineering Biology, Yale University, New Haven, Connecticut 06520, USA; 3 Department of Mechanical Engineering & Materials Science, Yale University, New Haven, Connecticut 06520, USA; 4 Benjamin Levich Institute and Physics Department, City College of New York, New York, New York 10031, USA; 5 Department of Applied Physics, Yale University, New Haven, Connecticut 06520, USA

## Abstract

During epithelial wound healing, cell morphology near the healed wound and the healing rate vary strongly among different developmental stages even for a single species like *Drosophila*. We develop deformable particle (DP) model simulations to understand how variations in cell mechanics give rise to distinct wound closure phenotypes in the *Drosophila* embryonic ectoderm and larval wing disc epithelium. We find that plastic deformation of the cell membrane can generate large changes in cell shape consistent with wound closure in the embryonic ectoderm. Our results show that the embryonic ectoderm is best described by cell membranes with an elasto-plastic response, whereas the larval wing disc is best described by cell membranes with an exclusively elastic response. By varying the mechanical response of cell membranes in DP simulations, we recapitulate the wound closure behavior of both the embryonic ectoderm and the larval wing disc.

In response to wounding, epithelia carry out complex chemical and physical processes to restore tissue integrity. Epithelial wound healing has been studied in numerous species, including *Drosophila*, zebrafish, and humans [1–7]. Even within a single species, the healing process varies with developmental stage [3,8]. In later stages, wound healing is slower, requires smaller changes in cell shape, and causes more scarring, which have been attributed to differences in chemical signaling, such as heightened inflammatory response [8–10]. However, physical mechanisms, such as force transmission through cell junctions and collective cell motion, have also been shown to influence wound healing [11–13]. An important driving force for wound closure across many developmental stages and species is the actomyosin purse string that forms around the wound [1,2,8,12,14]. Cell shape changes [15–17] are another physical mechanism that can affect the dynamics of wound healing in epithelial tissues. An important open question is determining how these *physical* mechanisms influence wound closure in different developmental stages.

Previous computational models of wound closure have investigated contributions from substrate mechanical properties, active driving forces, and tissue tension [3,12,18,19]. These models assume that cell membranes only respond elastically to deformation, ignoring viscoelastic and plastic response [20–22]. However, recent experimental studies have shown that irreversible cell shape changes [23–26] are necessary for cell stress relaxation and tissue remodeling. Neglecting cell membrane plasticity can give rise to unrealistically large stresses when significant cell shape changes are required for wound closure. It is therefore important to understand the role of viscoelastic and plastic response of cell membranes during wound closure.

*In vivo* studies of wound closure in late-stage *Drosophila* embryonic ectoderm and late third-instar *Drosophila* larval wing disc epithelium have found that cells near healed embryo wounds are elongated relative to those near healed wing disc wounds [3] [see Figs. 1(c) and 1(d)]. Embryo wounds close at a rate of ≈6.2 μm^2^/minute with cell shape changes of more than 30% near the wound, whereas wing disc wounds close at a rate of ≈0.7 μm^2^/minute with cell shape changes of less than 10% near the wound [see Figs. 1(a) and 1(b), plotted from experimental data in Ref. [3]]. The findings of Ref. [3] employ a vertex model approach [27] to predict that greater cell intercalation rates lead to increased wound closure speed. This prediction leads to an open question of how, relative to wing disc wounds, embryo wounds have lower intercalation rates yet heal more quickly [3]. To address this question, we propose that embryo ectodermal cells can rapidly remodel their membranes to sustain greater cell shape changes, which leads to faster wound closure rates than in the wing disc epithelium.

We carry out numerical simulations of the deformable particle (DP) model to explore the relationship between cell mechanical properties and wound closure phenotypes. We vary the degree of cell shape plasticity and determine the resulting effects on wound closure rate and cell shape deformation. We compare our simulation results to measurements of cell shape changes and wound closure rates from wounding experiments in embryonic and larval wing disc epithelia [3]. Our results suggest that cell shape plasticity is essential to achieve cell shape changes observed during embryo wound closure. Moreover, plasticity allows for faster wound closure rates in embryos compared to those in wing discs.

Since epithelial wound healing primarily involves in-plane motion, we consider a two-dimensional DP model for wound closure. The DP model has been studied recently in two and three dimensions, and DPs have been previously used to describe jamming and clogging of emulsion droplets and tissue morphogenesis [28–32]. The strengths of the DP model include the ability to describe both confluent and nonconfluent cell monolayers, both faceted and curved cell surfaces, and enable modeling both repulsive and cohesive intercellular forces. The shape energy [28] for each cell i is

(1) $$U_{\text{shape,}i}=\frac{k_{a}}{2}{\left(a_{i}-a_{0}\right)}^{2}+\frac{k_{l}}{2}\sum_{\alpha =1}^{N_{v}}{\left(l_{\alpha i}-l_{0\alpha i}\right)}^{2}+U_{b,i}.$$

Each cell is represented by a polygon with $N_{v}$ vertices (labeled by α), membrane bond vectors ${\vec{l}}_{\alpha i}={\vec{r}}_{\alpha i}-{\vec{r}}_{\left(\alpha -1\right)i}$, vertex positions ${\vec{r}}_{\alpha i}=\left(x_{\alpha i},y_{\alpha i}\right)$, equilibrium area $a_{0}$, and equilibrium intervertex membrane length $l_{0\alpha i}$. The area stiffness spring constant $k_{a}$ and membrane length spring constant $k_{l}$ penalize deviations of the cell area $a_{i}$ from $a_{0}$ and membrane length $l_{\alpha i}$ from $l_{0\alpha i}$ [see Fig. 2(a)]. We quantify cell shape using the shape parameter $A_{i}=p_{i}^{2}/4\pi a$, where $p_{i}$ is the perimeter of cell i and $A_{i}⩾1$. The bending energy

(2) $$U_{b,i}=\frac{k_{b}}{2}\sum_{\alpha =1}^{N_{v}}\theta_{\alpha i}^{2}$$

determines the energy cost of membrane curvature for cell i, where $k_{b}$ is the membrane bending rigidity and $\theta_{\alpha i}$ is the angle between ${\vec{l}}_{\alpha i}$ and ${\vec{l}}_{\left(\alpha -1\right)i}$.

The cells interact through the pair potential $U_{\mathrm{int}}$, which is a function of the distances between each vertex and nearby membrane segments on neighboring cells. $U_{\mathrm{int}}$ includes soft-core repulsion and short-range attraction with a variable well depth. We calculate the distance

(3) $$d_{\alpha i,\beta j}=\frac{\left|\left(x_{\left(\beta -1\right)j}-x_{\beta j}\right)\left(y_{\beta j}-y_{\alpha i}\right)-\left(x_{\beta j}-x_{\alpha i}\right)\left(y_{\left(\beta -1\right)j}-y_{\beta j}\right)\right|}{\left|{\vec{r}}_{\beta j}-{\vec{r}}_{\left(\beta -1\right)j}\right|}$$

between each vertex α on cell i and membrane segment ${\vec{l}}_{\beta j}$ on cell j as shown in Fig. 2(b). Having intercellular interactions that are only a function of $d_{\alpha i,\beta j}$ results in smooth sliding adhesion by eliminating components of the force on vertex α on cell i from interactions with cell j that are parallel to ${\vec{l}}_{\beta j}$ [See the definition of $U_{\mathrm{int}}$ in Eq. (S1) and the simulation parameters in Table S1 in the Supplemental Material [33].] The total energy $U_{DP}$ of a monolayer of N cells is

(4) $$U_{DP}=\sum_{i=1}^{N}U_{\text{shape,}i}+\sum_{i>j}^{N}\sum_{\alpha >\beta }^{N_{v}}U_{\mathrm{int}}\left(d_{\alpha i,\beta j}\right).$$

Animal cell membranes possess solid-like mechanical response on short and intermediate time scales, and are capable of stretching, bending, and transmitting forces [34,35]. To describe the viscoelasticity of the cell membrane, we model the membrane segments as springs that remodel their rest lengths in response to stress [Eq. (5)], similar in approach to models of irreversible deformation of the cytoskeleton and cell junctions [24,26,36]. We use rest-length remodeling to describe the net result of membrane stress relaxation processes, such as actin cortex remodeling, membrane folding and unfolding via caveolae, and vesicle trafficking via endocytosis and exocytosis [26,37,38]. We assume that the membrane segment rest length $l_{0\alpha i}$ obeys

(5) $$\frac{dl_{0\alpha i}}{dt}=-\frac{k_{l}}{\eta }\left(l_{0\alpha i}-l_{\alpha i}\right),$$

with damping coefficient η. The plastic relaxation timescale $\tau =\eta \text{/}k_{l}$ controls the membrane remodeling rate. In Fig. 2(c) we show a compression test of duration T on a single cell with an elastic $\left(\tau \text{/}T\gg 1\right)$ and plastic membrane $\left(\tau \text{/}T\ll 1\right)$. Cells with elastic membranes recover their undeformed shape, whereas cells with plastic membranes do not. By varying τ, we describe cells with different degrees of elasto-plasticity.

The wound simulations are conducted using overdamped equations of motion [Eq. (S5)], which are commonly used to model cell dynamics in the viscous extracellular environment [39,40]. We do not model explicit chemical signaling, and instead capture the biomechanical response that results from these chemical signals. Wounds are simulated by first generating a nearly confluent cell monolayer with A=1.2, similar to A of embryo and larval wing disc cells. We then remove the central cells from the monolayer, resulting in wounds similar in size to those in laser ablation experiments on epithelial monolayers [3]. In these simulations, we focus on the purse-string (PS) mechanism for wound closure. While embryonic wound healing features both PS and protrusive crawling activity [2,3], our simulations with only PS activity can predict the differences in embryonic and larval wing disc wound closure.

We define the PS as a collection of $N_{p}$ vertices along the wound boundary as shown in Fig. 2(d). Each PS vertex at position ${\vec{r}}_{p}\left(t\right)$ is initially coincident with a wound-adjacent DP vertex at ${\vec{r}}_{d}\left(t\right)$, such that ${\vec{r}}_{p}\left(0\right)={\vec{r}}_{d}\left(0\right)$. The two vertices are bonded by a spring with stiffness $k_{p}$, length $l=\left|{\vec{r}}_{p}-{\vec{r}}_{d}\right|$, and yield length $l_{y}$. For each PS vertex, the interaction energy is

(6) $$U_{PS}=\frac{k_{p}}{2}{\left[\min\left(l,\frac{l_{y}}{2}\right)\right]}^{2}\Theta \left(l_{y}-l\right),$$

where $\Theta \left(\cdot \right)$ is the Heaviside step function, and $U_{PS}$ saturates when $l\;⩾\;l_{y}\text{/}2$ and vanishes when $l>l_{y}$. Including $l_{y}$ ensures that PS vertices only interact with membrane segments near the wound. The PS contracts linearly in time t with constriction rate ω. Adjacent PS vertices are connected by springs with stiffness $k_{ps}=k_{l}$ and rest length

(7) $$\lambda_{0}\left(t\right)=\lambda_{0}\left(0\right)-\frac{\omega }{N_{p}},$$

which causes the PS to constrict over time. $\lambda_{0}\left(0\right)$) is chosen for each PS segment such that there is no initial tension, i.e., $\lambda_{0}\left(0\right)=l_{\alpha i}$.

To compare the wound closure simulation results with those from experiments, we analyze confocal microscopy images of wound closure in embryo and wing disc epithelia from Ref. [3] (see Figs. S3 and S4 in the Supplemental Material [33]). We convert simulation units to physical units using estimates of the adhesive force between two cells $f_{adh}\left(\approx 1\;\text{nN}\right)$ [41], cell area ($a_{0}\approx 25\;{\mu \text{m}}^{2}$ for embryo ectoderm and $a_{0}\approx 16\;{\mu \text{m}}^{2}$ for wing disc epithelium), and PS constriction rate $\left(\omega \approx 0.3\;\mu \text{m/s}\right)$ [42,43]. For example, the plastic relaxation time τ and cell bulk modulus B can be expressed in physical units as

(8) $$\tau =\tau^{*}\sqrt{a_{0}}/\omega ,$$

(9) $$B=k_{a}^{*}\frac{f_{adh}}{a_{0}},$$

where $\tau^{*}$ is the dimensionless plastic relaxation time and $k_{a}^{*}$ is the dimensionless area stiffness spring constant.

By varying τ and using realistic values of B, the wound closure simulations can recapitulate cell shape changes near the wound $\Delta A=A-A\left(0\right)$ [where $A\left(0\right)\approx 1.2$] and closure rates dA/dt that mimic those for embryo and wing disc wound closure. In Fig. 3 we show A of cells in the healed tissue that were adjacent to the wound boundary and dA/dt as a function of B and τ. Increasing cell membrane plasticity (i.e., decreasing τ) significantly increases dA/dt and ΔA. Elastic-like cells only achieve comparable ΔA to embryo cells when they are unrealistically soft $\left(B\;⩽\;0.04\;\text{kPa}\right)$, as experimental measurements of cell bulk moduli range from 0.3 to 2 kPa [44–46]. In a realistic range of B, elastic-like cells feature decreased dA/dt and smaller ΔA, with final shapes ranging from A=1.35to1.5 [Fig. 3(b)]. Small B alone does not result in the order of magnitude difference in dA/dt between embryo and wing disc wounds, suggesting that plasticity is essential to achieve ΔA and dA/dt found during embryo wound closure.

In elastic-like cells (i.e., τ>85 min in Fig. 3), increased B causes decreases in dA/dt and ΔA. This trend reverses in plastic-like cells (i.e., τ<20 min in Fig. 3). Increasing B dramatically decouples changes in membrane length from changes in area. Therefore, work done by $U_{p}$ strains membrane lengths significantly more than cell areas, enhancing ΔA when the membranes are plastic. We confirm this result by demonstrating that stiffer, plastic cells are more deformable than softer or more elastic cells, in simulations of a cell experiencing an extensile force dipole (see Fig. S5 in SM [33]).

We validate the DP model for wound closure by comparing the results of simulations in Fig. 3 to time-series data for the wound area and cell shape parameter in Figs. 1(a) and 1(b). The simulation parameters, except τ and B, are identical among different simulations in Fig. 3. We use softer and more plastic cells to model the embryo (red square) compared to those for the wing disc (black square). This choice reflects the expectation that cell stiffness increases with greater degree of cell differentiation [23,47], and the observation that embryo cell shapes deform faster and more severely than wing disc cell shapes. We find that our results for the time dependence of the wound area are consistent with the experimental data for the embryo and wing disc [Fig. 4(a)]. $A\left(t\right)$ in embryos matches the simulation results. In contrast, $A\left(t\right)$ in wing discs [Fig. 4(b)] requires an additional shape memory term in the membrane remodeling equation [cf. Eq. (5)],

(10) $$\frac{dl_{0\alpha i}}{dt}=-\frac{k_{l}}{\eta }\left[l_{0\alpha i}-l_{\alpha i}-\xi (l_{\alpha i}-l_{0\alpha i}(0)\right],$$

where $l_{0\alpha i}(0)$ is the segment length before wounding and ξ controls the timescale $\tau_{s}=\tau \text{/}\xi$ for cells to recover their original shape. We use ξ=0 for the embryo and ξ=0.1 for the wing disc, which leads to a better description of $A\left(t\right)$. This result suggests that more differentiated cells may have greater shape memory, perhaps to maintain their specialized functions.

Snapshots at several time points in Figs. 4(c) and 4(d) show how wound closure trajectories vary with B and τ. Soft and plastic cells form multicellular rosettes, which are common in embryonic wound healing and developmental processes [1,3,48]. Elastic-like cells exhibit less elongation towards the wound and fewer cell contacts near the wound, which is attributed to increased intercalation, akin to the wing disc wound closure process [3]. The difference in elongation between elastic- and plastic-like cells during wound closure arises due to differences in cell stress relaxation, as plastic cells can relax by elongation whereas elastic cells must relax by changing neighbors.

Deformable particle model simulations show that changes in cell stiffness and membrane plasticity lead to distinct wound closure phenotypes displayed in *Drosophila* embryo ectoderm and larval wing disc epithelium. The simulations take advantage of the deformable particle model’s ability to describe highly deformed cell shapes to incorporate membrane plasticity, which is not present in previous vertex model simulations of wound closure. By varying the cell bulk modulus B and plastic relaxation timescale τ, we find regimes that correspond to fast closure with significant cell shape changes, and slow closure with minor cell shape changes, which recapitulate the wound healing experiments on embryo and larval wing disc epithelia. We attribute the increased wound closure rates and greater cell shape changes to enhanced cell shape deformability, controlled by B and τ, in embryo cells relative to wing disc cells. These results show that the apparent paradox in Ref. [3] that embryo ectodermal wounds close more quickly than wing disc epithelial wounds can be resolved by considering a cell model with cell shape plasticity. Our model predicts that the key to explaining the differences in wound closure of *Drosophila* embryo and wing disc wounds is cell shape plasticity.

The correlation between rapid closure rate and distorted cell shapes during epithelial wound healing may suggest that earlier developmental stages prioritize fast healing at the expense of not maintaining the original tissue structure. Future work is necessary to determine whether cell shape plasticity is responsible for distinct patterns of tissue restructuring that occur during developmental processes, such as gastrulation and neurulation [49,50]. In addition, although the current study investigates cell shape changes from membrane surface remodeling, the role of cell volume plasticity is still unclear. Cell shape plasticity is the net result of surface area and volume plasticity, which are influenced by actin cortex remodeling and membrane reservoirs. Future experiments are necessary to investigate the separate contribution of these components to cell shape plasticity during wound closure.

## Supplementary Material

supplementary.pdf

## Figures and Tables

**FIG. 1.**
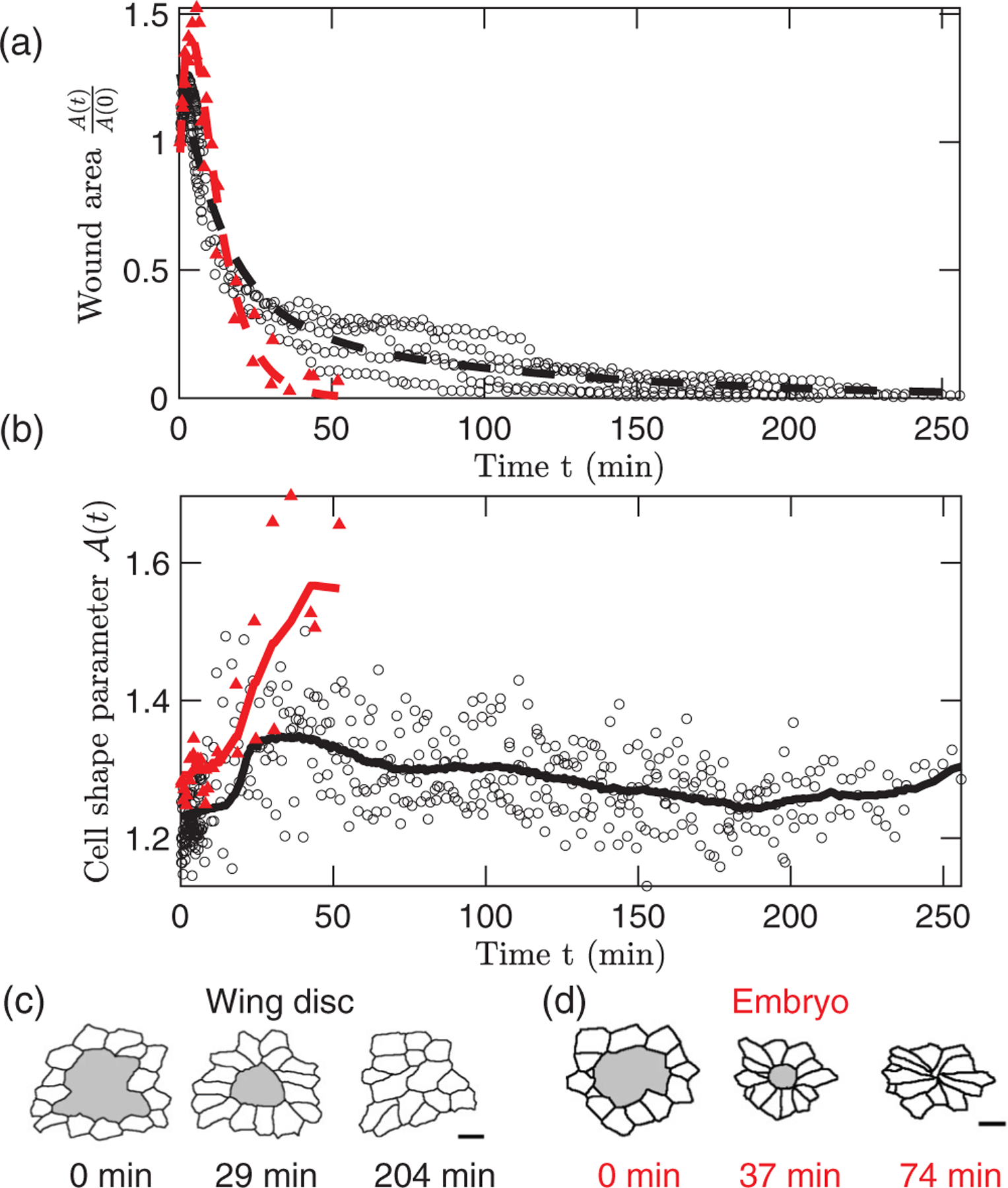
(a) Normalized wound area $A\left(t\right)\text{/}A\left(0\right)$ plotted as a function of time t during wound closure in wing discs (black circles) and embryos (filled red triangles), fit to a sum of two exponential functions (dashed lines). (b) Cell shape parameter $A\left(t\right)$ averaged over cells adjacent to the wound boundary and plotted vs time during wound closure for the same data in (a). We include a moving average of the data in (b) with a 20-minute window size (solid lines). The data for $A\left(t\right)\text{/}A\left(0\right)$ and $A\left(t\right)$ are from five wing disc wounds and two embryo wound experiments conducted in Ref. [3]. Example cell outlines reproduced from Ref. [3] are shown during wound closure for a single (c) wing disc and (d) embryo, with the wound shaded in gray. The scale bars are 3 μ in (c) and 5 μm in (d).

**FIG. 2.**
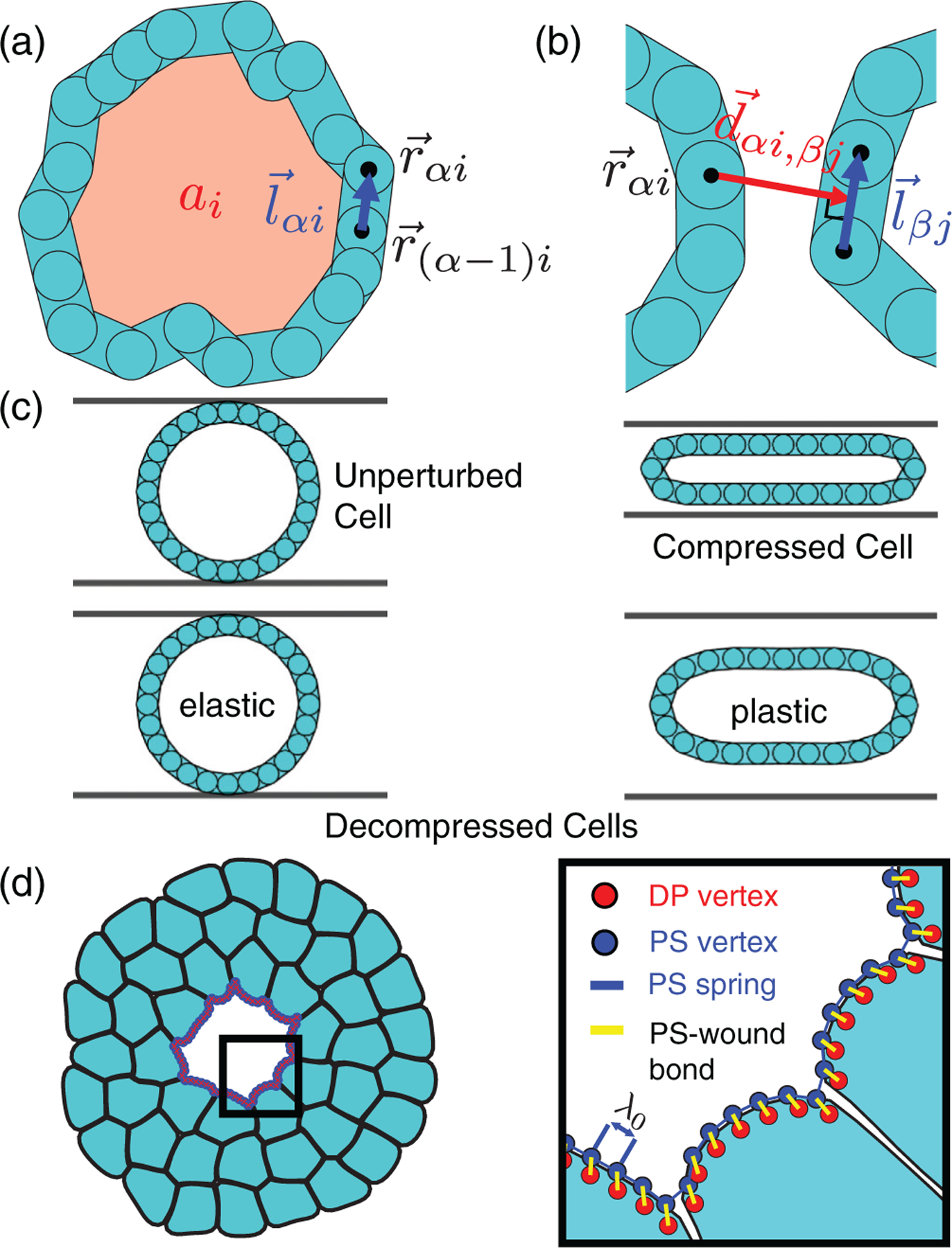
(a) Schematic of a deformable particle (or cell) with area $a_{i}$ and segment lengths $l_{\alpha i}=\left|{\vec{r}}_{\alpha i}-{\vec{r}}_{\left(\alpha -1\right)i}\right|$ representing the cell membrane. (b) The distance vector ${\vec{d}}_{\alpha i,\beta j}$ between vertex α on cell i and membrane segment ${\vec{l}}_{\beta j}$ on cell j has no component along ${\vec{l}}_{\beta j}$. (c) A cell with A=1 is uniaxially compressed. An elastic cell returns to its undeformed shape (left) after the strain is removed, whereas a plastic cell is permanently deformed (right). (d) A simulated wound is initialized as a cell monolayer, followed by removal of central cells such that the wound size is similar to those in Ref. [3]. Inset: Close-up of the purse string (PS), modeled as a collection of vertices (blue) along the edge of the wound (red). PS vertices are connected by springs (blue lines) with rest lengths $\lambda_{0}$, and each PS vertex is bonded to one DP vertex (yellow lines).

**FIG. 3.**
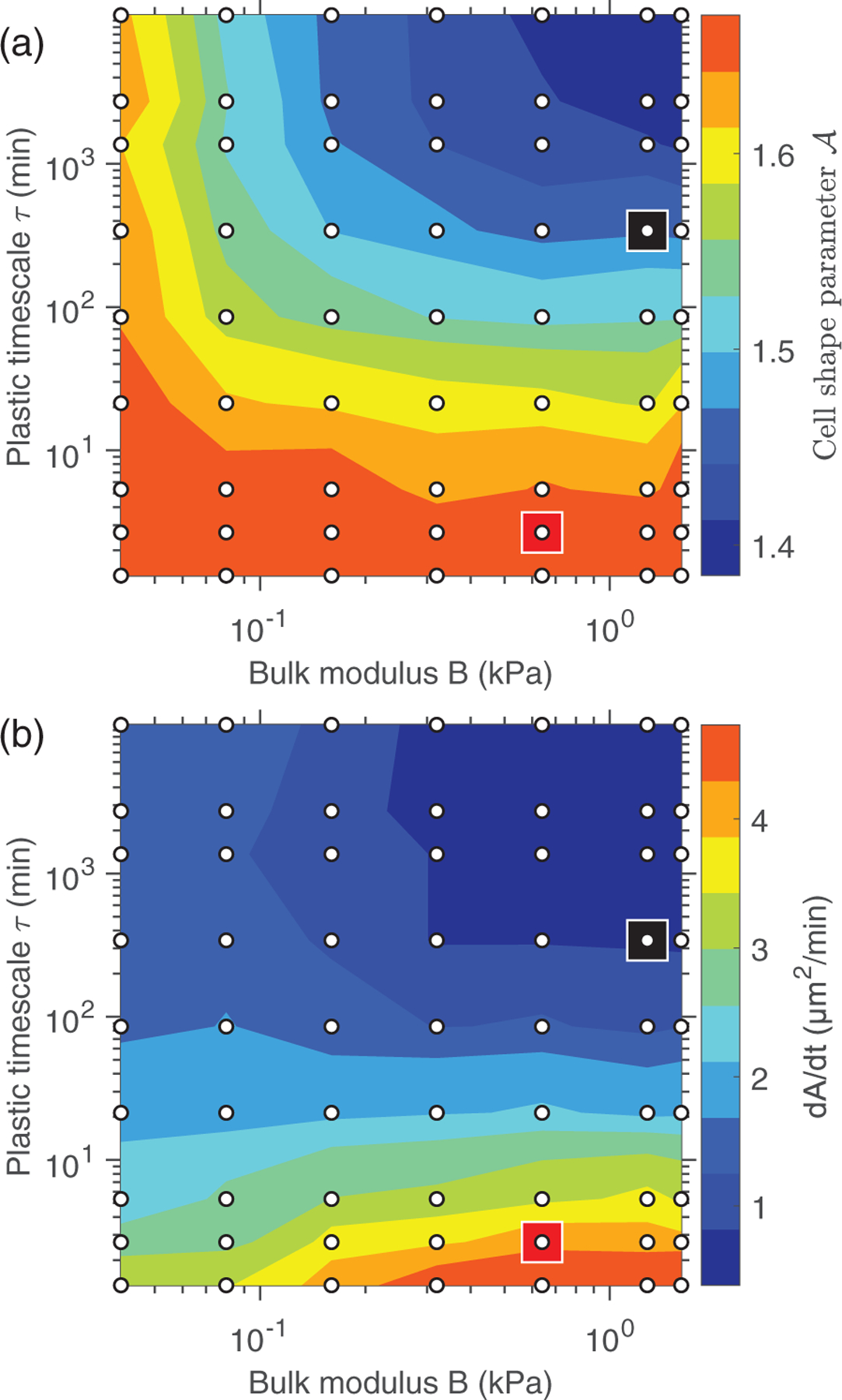
(a) Cell shape parameter A in the healed system plotted vs the cell bulk modulus B and plastic relaxation timescale τ. (b) Closure rate dA/dt plotted using the data in (a). Parameters indicated by the squares represent predictions for B and τ of embryo (red) and wing disc (black) cells. dA/dt is defined as 95% of the maximum wound area divided by the time it takes for the wound to shrink to 5% of its maximum size. Averaging at a given τ and β is performed over 25 simulations with different initial conditions. Simulations are carried out at B and τ given by the grid points, and contours are obtained via interpolation between grid points.

**FIG. 4.**
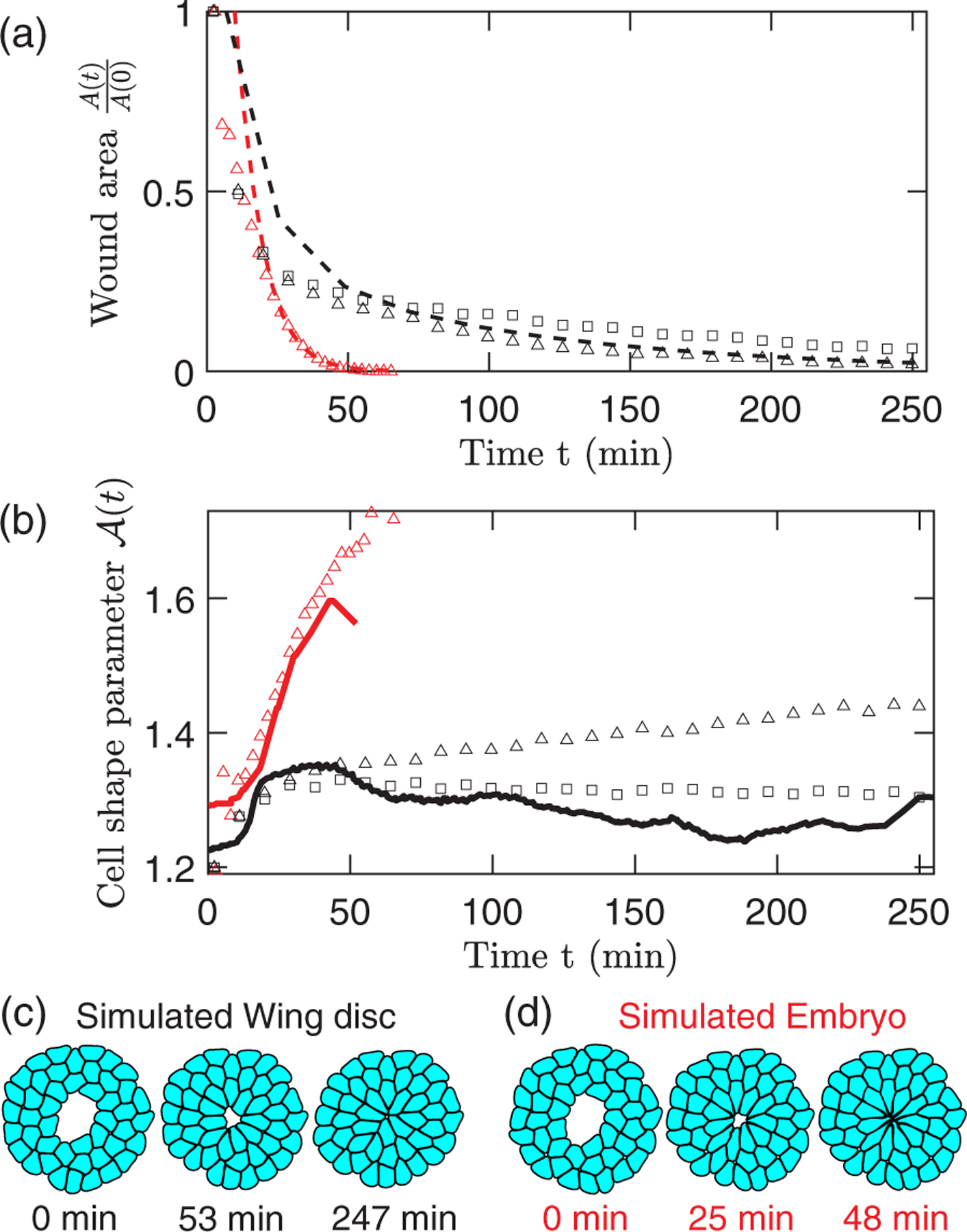
(a) Normalized wound area $A\left(t\right)\text{/}A\left(0\right)$ plotted vs time t for simulations of wound closure in embryo (red triangles) and wing disc cells (black triangles) using parameters in Fig. 3. (b) $A\left(t\right)$ averaged over cells adjacent to the wound for the simulations in (a). Solid lines indicate the moving average of $A\left(t\right)$ from experiments. Results from simulations using Eq. (10) are included in (a) and (b) to account for shape memory of wing disc cells (black squares). Snapshots of these simulations are shown for (c) embryo and (d) wing disc parameters.
