# Supplementary material for: Mechanical plasticity of cell membranes enhances epithelial wound closure
Source: Phys Rev Res. Author manuscript; Available in PMC 2025 Dec 25. (PMC12731943; doi:10.1103/physrevresearch.6.l012036)
Supplement: supplementary.pdf [file NIHMS2118852-supplement-supplementary_pdf.pdf]

Supplemental Material for
Mechanical Plasticity of Cell Membranes Enhances Epithelial Wound Closure

Andrew T. Ton,[1,2] Arthur K. MacKeith,[3,2] Mark D. Shattuck,[4] and Corey S. O'Hern[3,1,5,2]

[1]*Department of Physics, Yale University, New Haven, Connecticut 06520, USA*
[2]*Integrated Graduate Program in Physical and Engineering Biology, Yale University, New Haven, Connecticut 06520, USA*
[3]*Department of Mechanical Engineering & Materials Science, Yale University, New Haven, Connecticut 06520, USA*
[4]*Benjamin Levich Institute and Physics Department, The City College of New York, New York, New York 10031, USA*
[5]*Department of Applied Physics, Yale University, New Haven, Connecticut 06520, USA*
(Dated: December 10, 2023)

The supplemental material includes seven sections that provide additional details concerning the deformable particle simulations and analyses of the experimental data of wound closure in epithelial monolayers. In Section I, we describe the intercellular forces, initialization of the cell monolayer, generation of the wound, equations of motion, and tracking of the wound boundary over time in the numerical simulations of wound closure. In Section II, we provide a novel method for calculating smooth intercellular forces in the deformable particle model simulations. In Section III, we describe the cell segmentation, cell shape parameter calculations, and error estimation from the analyses of confocal microscopy images of wound healing in epithelial tissues in *Drosophila* [1]. In Sections IV and V, we relate the cell bulk modulus to the deformable particle area stiffness, and express the bulk modulus and other simulation quantities in physical units. In Section VI, we study the deformation of a cell experiencing an extensile force dipole to give further evidence that a larger bulk modulus leads to enhanced shape changes in plastic cells. In Section VII, we discuss multiple contributing factors to the overall process of cell shape plasticity.

I. WOUND CLOSURE SIMULATION PROTOCOL

We model cell monolayers as nearly confluent packings of deformable particles. Each deformable particle (cell) i obeys the shape energy in Eq. 1 in the main text and interacts with other cells through vertex-segment forces between vertex α on cell i and segment $\vec{l}_{\beta j}$ on cell j as shown in Fig. 2 in the main text. The intercellular forces can be derived using the following pair potential:

$$U_{int} = \sum_{i>j} \sum_{\alpha>\beta} u_{int}(d_{\alpha i,\beta j}), \tag{S1}$$

where

$$u_{int}(d) = \begin{cases} \epsilon \left[(1 - \frac{d}{\sigma})^2 - \frac{l_1 l_2}{\sigma^2} \right], & 0 < \frac{d}{\sigma} < 1 + \frac{l_1}{\sigma} \\ \epsilon \frac{l_1}{l_1 - l_2} \left[1 + \frac{l_2}{\sigma} - \frac{d}{\sigma} \right]^2, & 1 + \frac{l_1}{\sigma} < \frac{d}{\sigma} < 1 + \frac{l_2}{\sigma}, \end{cases} \tag{S2}$$

ϵ is the cell membrane interaction strength, $d \equiv d_{\alpha i,\beta j}$ is the vertex-segment distance, σ is half the membrane width, $l_2 = 0.3\sigma$ sets the attractive range, l_1 controls the membrane attraction strength, and $l_1 < l_2$. The vertex-segment pair potential u_{int} and the force $f_{int} = -\partial u_{int}/\partial d$ are plotted against d/σ in Fig. S1. See Table S1 for a list of default parameters and other quantities used in the simulations.

To generate unwounded cell monolayers, we first place cells with random initial positions within a circular boundary at an initial packing fraction $\phi_0 = 0.78$, and then compress the system in small packing fraction increments $\Delta\phi = 0.005$ until $\phi = 0.92$. After each compression step, we use the FIRE algorithm [2] to minimize the total potential energy of the cells within the circular boundary:

$$U'_{DP} = \sum_{i}^{N} U_{shape,i} + U_{rep} + U_{cb}, \tag{S3}$$

where U_{rep} is equal to U_{int} with $l_1 = 0$ and

$$U_{cb} = \begin{cases} \epsilon_{cb} \sum_{i}^{N} \sum_{\alpha}^{N_V} \left(1 - \frac{|r_{\alpha i} - R|}{\sigma} \right)^2, & |r_{\alpha i} - R| < \sigma \\ 0, & \text{otherwise.} \end{cases} \tag{S4}$$

Here, $r_{\alpha i}$ is the distance between a vertex α on cell i and the center of the circular boundary of radius R. We set $\epsilon_{cb} = \epsilon$ as the strength of the interaction energy between the circular boundary and the vertices.

The resulting packings of deformable particles with $\mathcal{A} = 1.2$ and $\phi = 0.92$ serve as initial conditions for the numerical simulations of wound closure. First, we add cell-cell adhesion to the packings by setting $l_1/\sigma = 0.1$ and carry out constant energy dynamics without the circular boundary for a total time $\sim \sqrt{a_0}/\omega$ to allow the cells to explore intercellular gaps. We then minimize the total potential energy U_{DP} (Eq. 4 in the main text) using an overdamped equation of motion, where each vertex α on cell i obeys

$$\frac{d\vec{r}_{\alpha i}}{dt} = \frac{\vec{f}_{\alpha i}}{b}. \tag{S5}$$

Here, $\vec{f}_{\alpha i}$ is the total force on the vertex i, and b is the damping coefficient. Overdamped dynamics are frequently employed in active particle models [3] to balance the input of energy due to activity, which corresponds to the purse-string contraction in this model. The effects of the cells' environment on cell dynamics are commonly modeled using a single damping coefficient [3, 4], as we have done in Equation S5.

We then introduce a wound by removing 5 central cells in the packing, define the purse-string (PS) on the wound boundary, and integrate Eq. S5 using a modified velocity-Verlet algorithm with timestep $dt^* = 0.05/\sqrt{k_a^*}$. We record cell properties, such as the cell shape parameter $\mathcal{A}$, and wound area A throughout the course of the simulation. The simulated wound boundary is tracked over time by monitoring vertex-vertex contacts and determining the largest cluster of vertices near the center of the tissue using the Newman-Ziff union-find algorithm [5]. We terminate the wound closure simulation when the wound area satisfies $A < 10^{-2} a_0$. In both the numerical simulations and image analyses, a cell is considered wound-adjacent if it is within $\sqrt{a_0/\pi}$ of the wound boundary.

II. SMOOTH SLIDING INTERCELLULAR FORCES

Intercellular forces are bumpy in the deformable particle model when the intercellular potential is a function of the vertex-vertex distances. We develop a novel method (within the deformable particle model) to compute *smooth* (frictionless) sliding intercellular forces. To do this, we assume that the intercellular pair potential U_{int} is only a function of the closest distance $d_{\alpha i,\beta j}$ between vertex α on cell i and line segment $\vec{l}_{\beta j}$ on cell j (Eq. 3 in the main text), which ensures that there is no component of the intercellular force tangential to the membrane at $\vec{l}_{\beta j}$ as shown in Fig. S2a. This force law mimics smooth surfaces that consist of connected rectangles (cyan) and wedges (blue) in Fig. S2b. Because $d_{\alpha i,\beta j}$ is a function of $\vec{r}_{\alpha i}$, $\vec{r}_{\beta j}$, and $\vec{r}_{(\beta-1)j}$, forces computed using the pair potential $u_{int}(d_{\alpha i,\beta j})$ will affect the dynamics of all three vertices α, β, and $\beta - 1$.

For interaction potentials that depend on the closest distance between a point and a line segment, one must determine whether there are discontinuities in the force that can occur when the closest distance changes discontinuously even though a vertex or line segment moves by an infinitesimal amount. We consider two cases, concave and convex sections of the cell membrane, classified by the interior angle θ defined by three successive vertices. In the convex case, $\theta < \pi$ (Fig. S2c), a vertex that overlaps with the membrane surface at $\vec{r}_{contact}$ can slide along the surface with a continuous vertex-segment distance $d_{\alpha i,\beta j}$ to the line segment $\vec{l}_{\beta j}$. In the concave case, $\theta > \pi$ (Fig. S2d), a vertex on a similar trajectory will experience a discontinuity in the vertex-segment distance, since the concave surface (Fig. S2b) lacks a wedge where there is an overlap of the two rectangles. One method to remove this discontinuity is to add a wedge-shaped patch, shown as a red grid in Fig. S2e. Within the wedge-shaped region, a vertex α overlapping with the membrane surface $l_{\beta j}$ at $\vec{r}_{contact}$ incurs an additional force

$$f_{patch} = +\frac{\partial u_{int}}{\partial d_{\alpha i,\beta j}}, \tag{S6}$$

such that in the patch region, f_{patch} provides an equal and opposite force to offset the discontinuity in $f_{int}(d_{\alpha i,\beta j})$ (Fig. S1b) that occurs when the vertex α enters the patch region.

III. IMAGE ANALYSIS

To measure the cell shape parameters and wound area over time of the wounded embryo and wing disc epithelia, we analyze segmented images of the wound closure process from Ref. [1]. We use the segmented cell boundaries in the 5 wing disc wounds found in Ref. [1]. For the 2 embryo wounds in Ref. [1], we perform our own segmentation procedure by first taking a maximum intensity projection (Fig. S3a) along the z-axis of confocal microscope z-stack time-series images. Next, we use *Tissue Analyzer* [6], an ImageJ plugin for segmentation of single-layered epithelia, to obtain a first pass segmentation of the cells and wound (Fig. S3b). Then we make manual corrections to account for under- and over-segmented regions near the wound, which yields a binary image of the cell boundaries (Fig. S3c). We employ *regionprops* in MATLAB R2022a, Update 5 (Fig. S3d-e) to calculate the area and perimeter of each unique segmented region, which we use to report the wound areas and cell shape parameters $\mathcal{A}$ for each time point.

To generate error estimates for the cell shape parameter measurements, we carry out a similar process on synthetic data. We use 26 test shapes: ellipses with eccentricities 0.996, 0.987, 0.968, 0.933, 0.872, 0.768, 0.586, and 0 (circle), polygons with 3 to 12 sides, and 8 different 7-sided concave shapes. A subset of the test shapes is shown in Fig. S4a. We generate images of these shapes with a range of resolutions (Fig. S4b) and compare the measured $\mathcal{A}$ to the true value $\mathcal{A}_t$ for each shape (Fig. S4c). We define the fractional error of $\mathcal{A}$ as

$$\delta\mathcal{A} = \frac{\mathcal{A}_t - \mathcal{A}}{\mathcal{A}_t}. \tag{S7}$$

For a cell with area a, we report an estimate of $\delta\mathcal{A}$ using the bounding fractional error $\mathcal{E}(a)$ as shown in Fig. S4c. $\mathcal{E}(a)$ is calculated by taking the maximum $\delta\mathcal{A}$ over all synthetic test shapes at each area, and storing the running maximum as a function of decreasing area. For each measurement of the cell shape parameter $\mathcal{A}$ for a cell with area a (px^2) in the embryo and wing disc wounding

experiments, we associate $\mathcal{E}(a)$ with the measurement error in $\mathcal{A}$.

We plot $\mathcal{A}(t)$ taking the variance-weighted mean over cells adjacent to the wound boundary (Fig. S3d), with error bars given by the standard error of this weighted mean. Onto the variance-weighted mean and error bars, we overlay the simulation results as in Fig. 4b in the main text to show that $\mathcal{A}(t)$, from simulations of the embryo and the wing disc using cells with shape memory, falls within the margin of error for the $\mathcal{A}(t)$ from the experimental measurements of the embryo and wing disc wounds.

IV. DERIVATION OF BULK MODULUS

The bulk modulus B of a single cell is related to the cell area stiffness k_a through Eq. 9 in the main text. To derive this relation, we start with the definition

$$B = -V\frac{dP}{dV},$$

where P is the pressure and V is the cell volume. Rearranging, we obtain

$$dP = -\frac{B}{V}dV,$$

and $P = B\log(V/V_0)$, where $V = V_0$ at zero pressure. The energy under isothermal compression is given by

$$U_c = \int PdV' = BV(\log\left(\frac{V}{V_0} - 1\right) + BV_0.$$

To second order in $V/V_0 - 1$, we obtain

$$U_c \approx \frac{1}{2}BV_0\left(\frac{V}{V_0} - 1\right)^2. \quad \text{(S8)}$$

Comparing Eq. S8 to the shape-energy function (Eq. 1 in the main text), the energy due to compression in two dimensions is given by

$$U_{DP,c} = \frac{1}{2}k_a a_0^2\left(\frac{a}{a_0} - 1\right)^2.$$

Assuming that the energy scale of volume changes is equal to that of area changes, and that $V_0 = a_0^{3/2}$,

$$B = k_a\sqrt{a_0}.$$

V. CONVERTING SIMULATION UNITS TO PHYSICAL UNITS

Simulation units can be converted into physical units using three physical quantities that set the mass, length, and time scales of the simulation. We are able to determine these scales using a choice of force, velocity, and area (See Table S1.). Atomic force microscopy can determine single cell forces [7], which allows us to estimate the unsticking force $f_{adh} = 1$ nN based on cohesion between Zebrafish embryo ectodermal cells [8]. We are unaware of any measurements of f_{adh} on *Drosophila* embryo ectoderm, and we assume that the measurements of f_{adh} on Zebrafish embryo ectoderm give a reasonable estimate. We choose $\omega = 0.3$ μm/sec based on a typical actin ring constriction rate [9, 10]. We find that typical *Drosophila* cell areas are $a_0 \sim 16$ μm^2 for the late-stage larval wing disc epithelium and ~ 25 μm^2 for the late-stage embryo ectoderm.

We define the unsticking force in our simulations by $f_{adh} = l_1\epsilon N_v/3\sigma^2$ with units of ϵ/σ. A factor of $N_v/6$ comes from the assumption that two cells are adhered to each other through 1/6 of their membranes on average, given that a cell has approximately 6 neighbors. The maximum vertex-vertex adhesion force in Eq. S1 is $2l_1\epsilon/\sigma^2$, again with units of ϵ/σ.

VI. STIFFNESS ENHANCES SHAPE CHANGE IN PLASTIC CELLS

To understand how greater stiffness can lead to enhanced shape change in plastic cells, we conduct simulations varying the plastic relaxation timescale τ and the cell area stiffness k_a of a single cell experiencing an extensile force dipole (Fig. S5a), i.e. the cell experiences two equal and opposite forces that generate net zero force. For elastic-like cells with large τ, we find that increasing k_a leads to a reduction in the final $\mathcal{A}$ (Fig. S5b), which matches the expectation that stiffer cells are less deformable. However, the trend reverses for plastic-like cells with small τ, as increasing k_a leads to an increase in the final $\mathcal{A}$. We find that stiff, plastic cells are able to lengthen their membranes without changing their area. In contrast, soft plastic cells increase their area when lengthening their membranes, which results in more modest shape changes. These results show that plastic cells become more deformable as they become stiffer, and corroborates a similar trend in Fig. 3 in the main text.

VII. ALTERNATIVE MECHANISMS INFLUENCING CELL SHAPE PLASTICITY

Cell shape plasticity, the property of cells allowing them to retain their new shapes after deformation, is the result of several mechanisms, which include actin cortex remodeling, caveolae acting as membrane reservoirs, and vesicle trafficking processes like exocytosis and endocytosis. These processes are involved in mechanoprotection, and allow the cell to modulate the cell membrane surface area in response to stress. In our model, a natural way to incorporate changes in cell membrane surface area due to membrane reservoirs and vesicle trafficking is to add plasticity in the membrane rest length. We describe

cell shape plasticity as membrane plasticity using Equation 5. A different model for cell shape plasticity could add plasticity in the equilibrium bending angle of each membrane segment, which would account for how actin cortex remodeling contributes to cell shape plasticity independently of membrane surface area relaxation processes. Since curvature is dependent on both membrane segment lengths and the angles between the segments, we note that membrane rest length plasticity also includes relaxation of membrane curvature.

**Simulation quantities**	**Symbol**	**Value**
**Embryo cell rest area**	$\mathbf{a_0}$	25 μm^2
**Wing disc cell rest area**	$\mathbf{a_0}$	16 μm^2
**Unsticking force**	$\mathbf{f_{adh}}$	1 nN [8]
**PS constriction rate**	$\boldsymbol{\omega}$	0.3 μm/sec [9, 10]
Numerical integration timestep	dt^*	$0.1 l_0^*(0)\sqrt{a_0^*/k_a^*}$
DP vertex damping coefficient	b^*	1 (damped)<br>0 (constant energy)
Number of vertices per DP	N_v^*	30
Cell area stiffness	k_a^*	0.25, 0.5, 1.0,. . .,256
Membrane length spring constant	k_l^*	1
Membrane bending rigidity	k_b^*	0.01
Cell rest area	a_0^*	1
Initial membrane segment rest length	$l_0^*(0)$	$\sqrt{\mathcal{A}4\pi a_0^*}/N_v$
Half membrane width	σ^*	$l_0^*(0)/2$
Membrane interaction energy	ϵ^*	1
Maximum vertex-vertex adhesion force	$2l_1^*$	0.2
Unsticking force	$f_{adh}^* = l_1^* N_v/3$	1
Plastic relaxation timescale	$\tau^* = \eta^*/k_l^*$	2.4, 4.8, 9.6,. . .,39322
PS-DP spring constant	k_p^*	4
PS-DP spring yield length	l_y^*	$4\sigma^*$
PS constriction rate	ω^*	1
Wound closure rate	$\omega\sigma dA^*/dt^*$	— (μm/s)
Cell shape parameter	$\mathcal{A}$	—

TABLE S1. Default parameters and other quantities used in the deformable particle simulations of wound closure. An asterisk denotes non-dimensional simulation units. Bolded quantities have physical units and are based on experimental measurements. If only one parameter value is listed, then the parameter is not varied in our simulation studies.

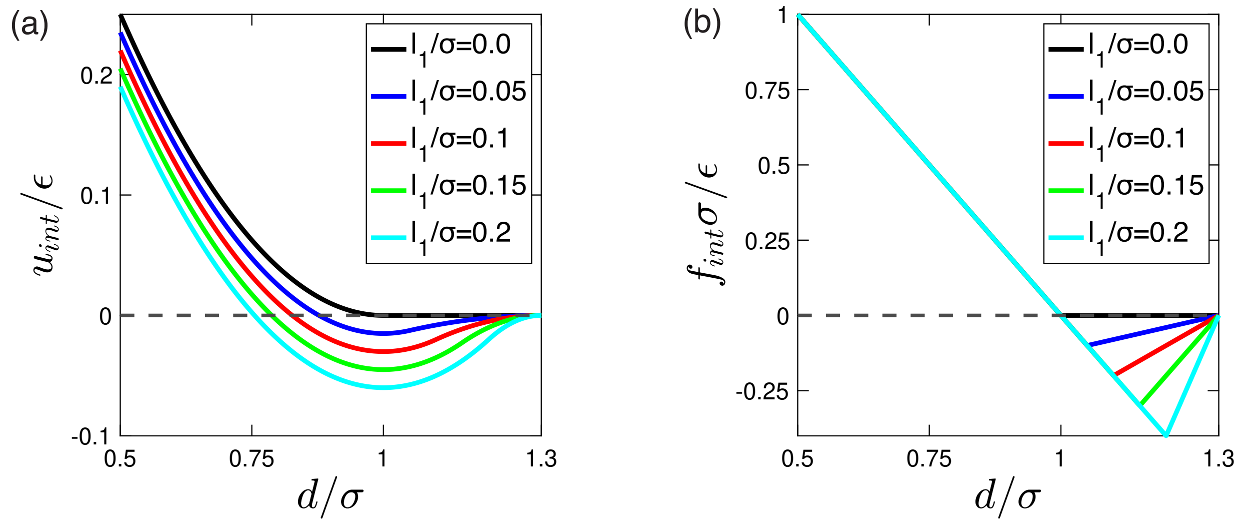

FIG. S1. (a) Vertex-segment pair potential energy u_{int} and (b) vertex-segment force f_{int} is plotted against the vertex-segment distance d for several values of the attraction strength l_1/σ. u_{int}, f_{int}, d, and l_1 are all nondimensionalized using the half membrane width σ and membrane interaction strength ϵ. l_2 is fixed at 0.3σ for all numerical simulations of wound closure.

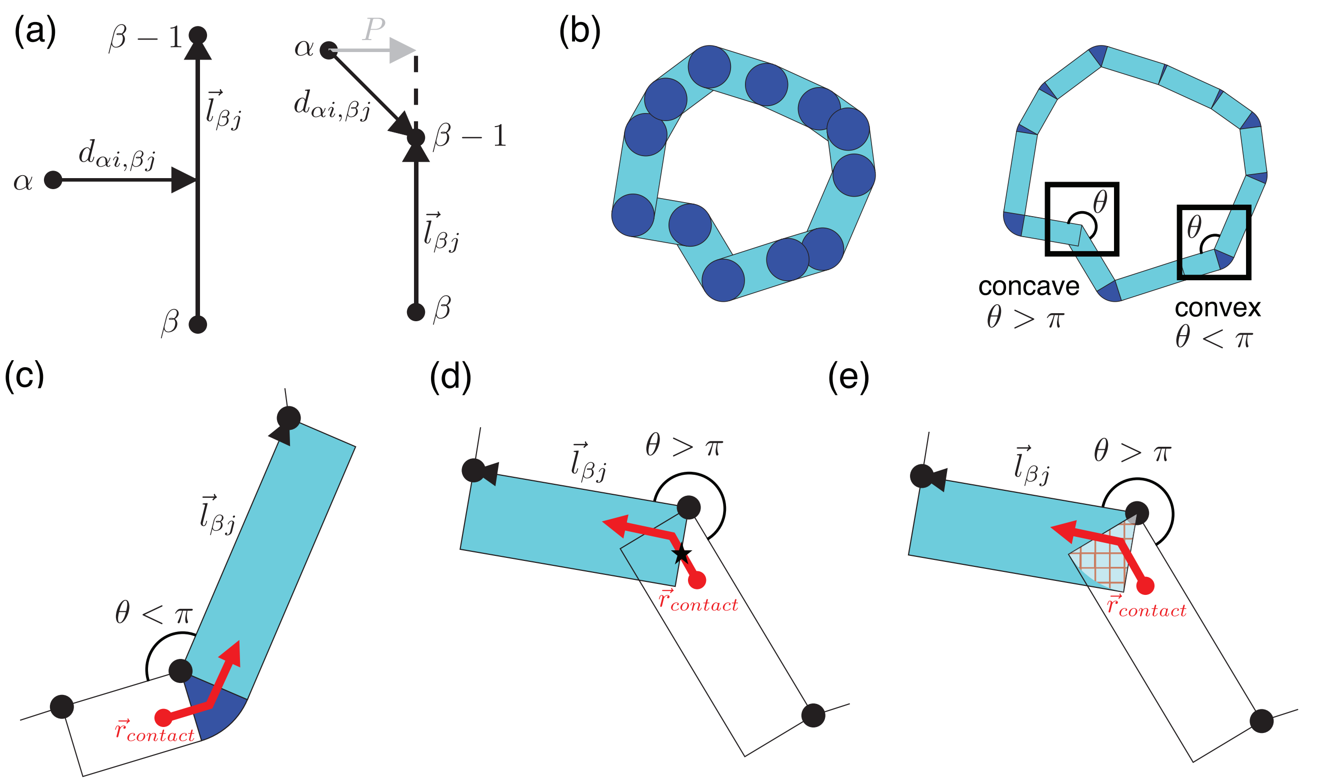

FIG. S2. (a) The vertex-segment distance $d_{\alpha i,\beta j}$ is the shortest distance from vertex α on cell i to line segment $\vec{l}_{\beta j}$ on cell j. If the projection P of $\vec{d}_{\alpha i,\beta j}$ to the segment $\vec{l}_{\beta j}$ (gray arrow) falls outside of $l_{\beta j}$, then $d_{\alpha i,\beta j}$ is the distance between vertex α on cell i and vertex β or $(\beta - 1)$ on cell j. (b) Each deformable particle (left) is modeled as a collection of vertices (dark blue circles) connected by line segments (cyan region). In the right image, we also show the exterior-facing half of the cell membrane of a "smooth" deformable particle, which consists of circulo-line segments (cyan) and vertex sectors (blue). Within a smooth deformable particle, the section around each vertex is considered concave when the angle the vertex makes with its two neighboring vertices has an interior angle $\theta > \pi$, and convex otherwise. (c) For locally convex geometries, when a vertex that overlaps the membrane surface at $\vec{r}_{contact}$ moves along the indicated trajectory (red line), the vertex-segment distance relative to $\vec{l}_{\beta j}$ is continuous. (d) For locally concave geometries, the vertex-segment distance (relative to $\vec{l}_{\beta j}$) for a vertex overlapping with the membrane along the trajectory $\vec{r}_{contact}$ (red line) undergoes a discontinuous jump when crossing the point indicated by the black star. (e) The wedge-shaped patch (red grid) indicates the region over which the force in Eq. S6 acts to remove the discontinuity in force that occurs in $\partial u_{int}/\partial d_{\alpha i,\beta j}$ in the case of locally concave membrane geometries. The wedge also removes a similar discontinuity relative to $l_{(\beta-1)j}$.

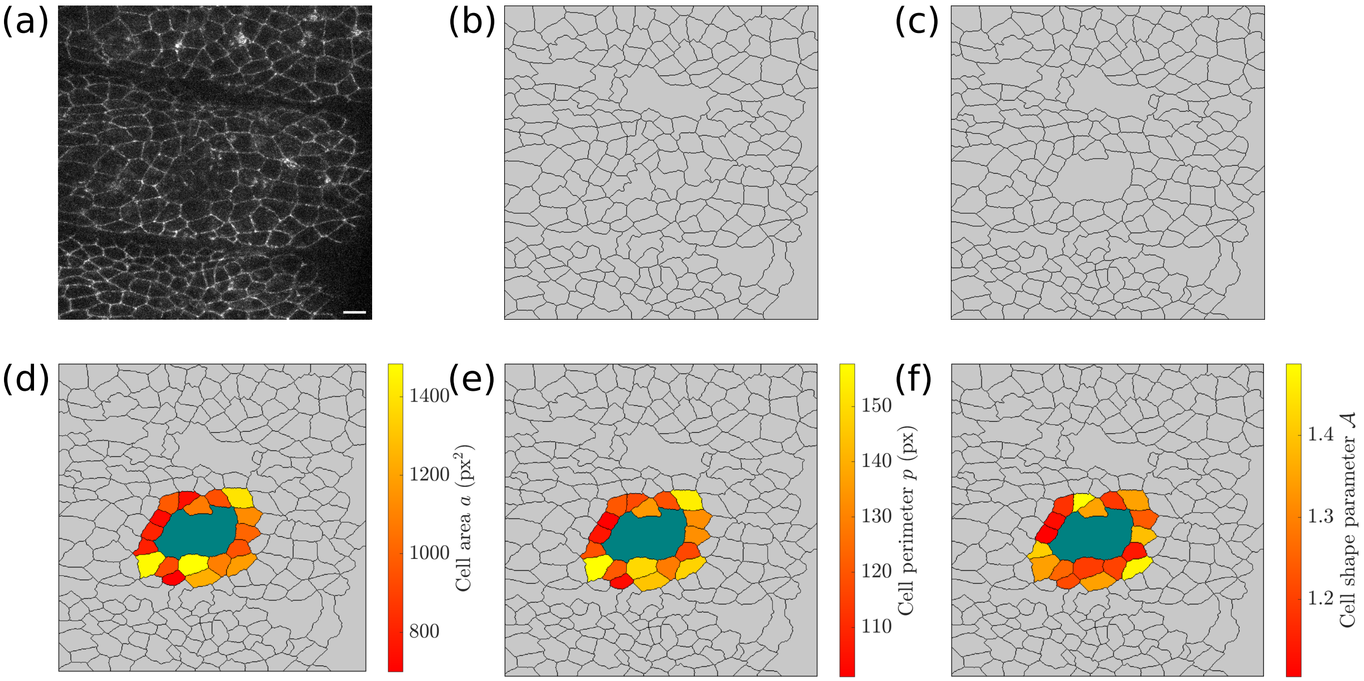

FIG. S3. (a) The first step in the image analysis pipeline is to transform raw confocal microscopy images into maximum intensity projections along the z-axis, shown here for a *Drosophila* embryo ectoderm immediately after wounding from Ref. [1]. The scale bar has width 10 μm. (b) We next perform automated cell boundary segmentation using Tissue Analyzer [6], an ImageJ plugin for segmentation of single-layered epithelia. (c) We make manual corrections to cell boundaries near the wound in each frame. From the segmented cell boundaries, we calculate (d) the cell area a and the wound area, (e) the cell perimeter p, and (f) the shape parameter $\mathcal{A}$ of cells adjacent to the wound. In this example, the wound area is 9048 px^2 or 157 μm^2.

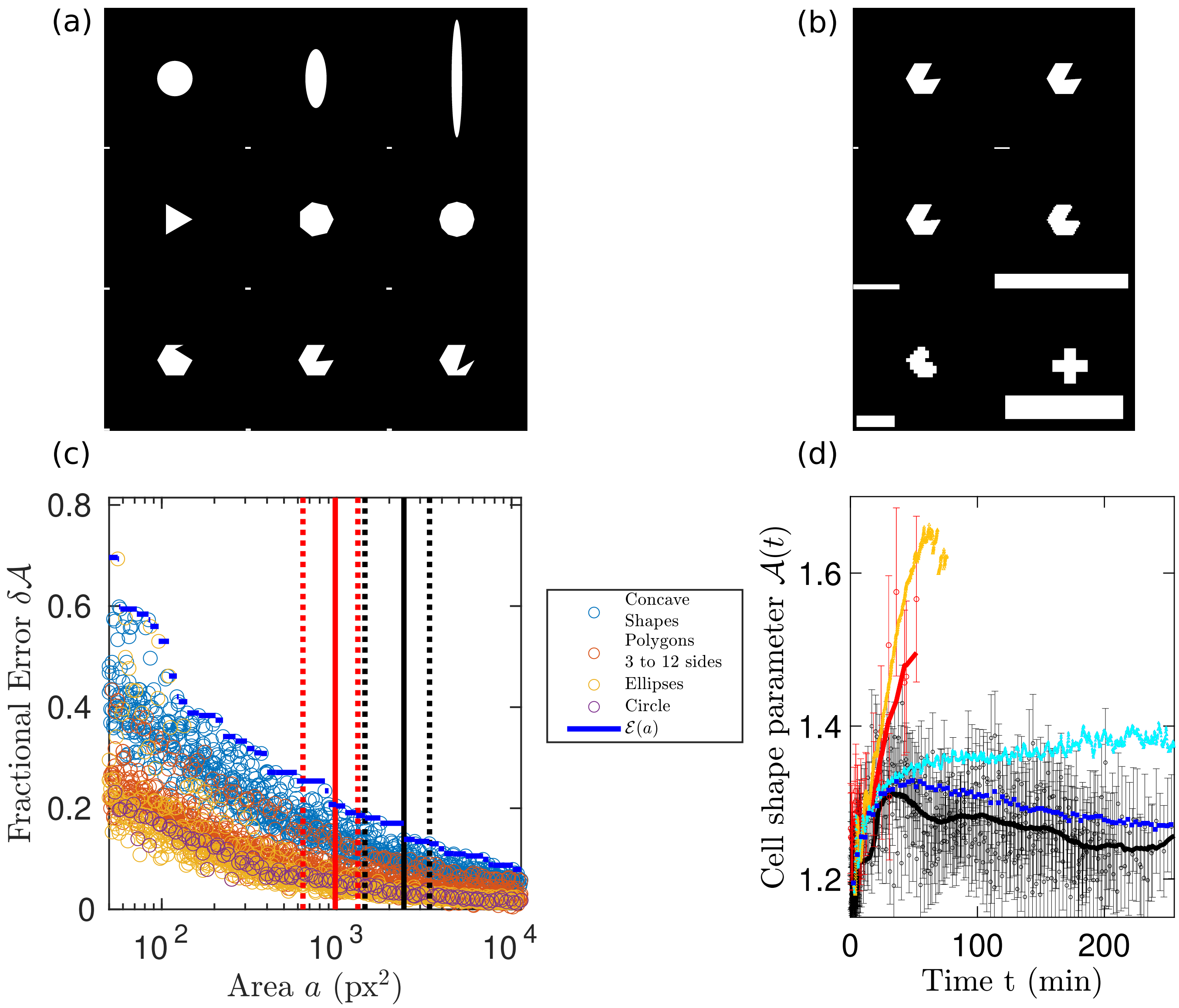

FIG. S4. (a) Synthetic data is shown at high resolution: From left to right, then top to bottom: ellipse with $e = 0$ (circle), ellipse with $e = 0.933$, ellipse with $e = 0.996$, triangle, heptagon, dodecagon, concave shape 1, 4, and 8. The scale bars to the lower left of each shape are 100 px wide. (b) A shape with an interior angle $\theta > \pi$ is shown at several resolutions. The scale bars to the lower left of the top four images are 100 px wide, and the scale bars in the bottom two are 10 px wide. The scale bar sizes in the images vary due to significant changes in the image resolutions. (c) The fractional error in $\mathcal{A}$ measured in synthetic data is plotted versus the number of pixels contained inside the shape over an experimentally relevant range. Estimate of the error in $\mathcal{A}$ as a function of the area of the shape in the synthetic images (blue line). The red and black solid lines represent the mean areas in the experimental data sets of embryo and wing disc cells, respectively, and the spacings between the dotted lines represent the standard deviations. (d) We plot $\mathcal{A}(t)$ for the embryo (red) and wing disc (black) experiments using the variance-weighted mean (open circles) over cells adjacent to the wound, where the variance is given by $\mathcal{E}^2(a)$. We include error bars using the standard error of the weighted mean. The solid lines are moving averages with the same window size as in Fig. 1b. We overlay the simulation results for the embryo (yellow triangles), the wing disc (cyan triangles), and the wing disc using cells with shape memory (blue squares), all of which are the same data as in Fig. 4 in the main text.

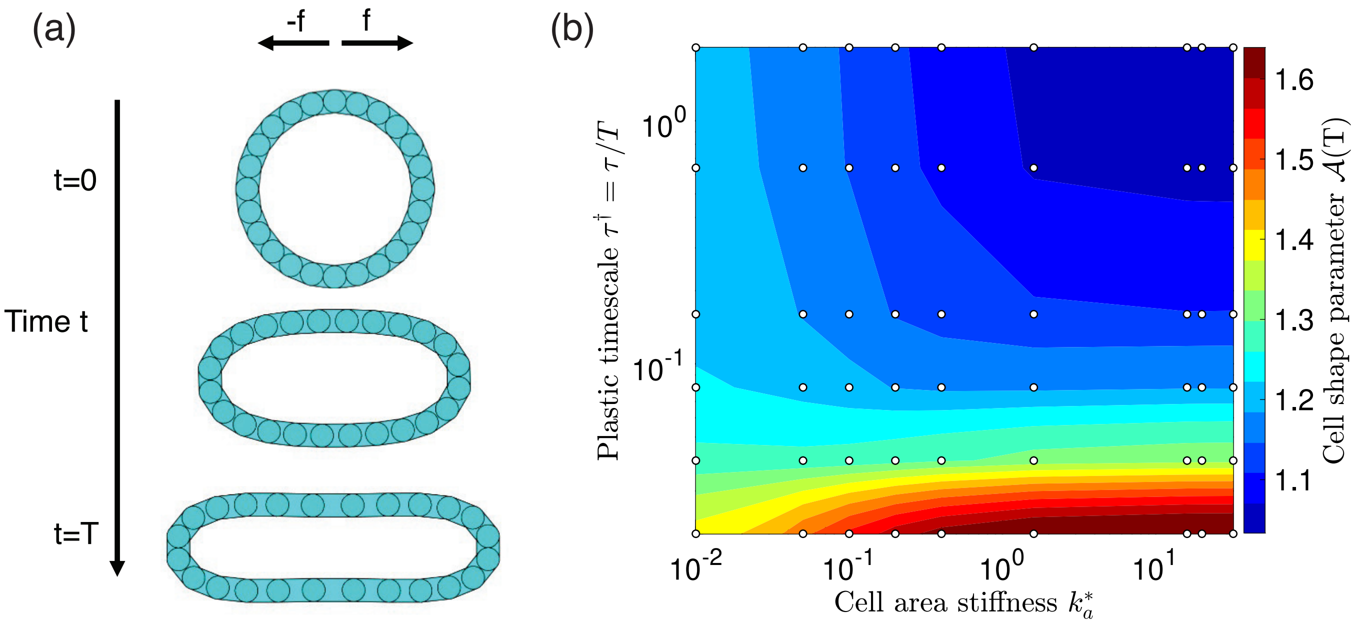

FIG. S5. (a) A deformable particle is shown with an extensile force dipole with magnitude f that generates elongation over time t. (b) Final cell shape $\mathcal{A}(T)$ of the extensile deformable particle as a function of the cell area stiffness k_a^* and the normalized plastic relaxation timescale $\tau^{\dagger} = \tau/T$, where T is the duration of the stretching simulation.

[1] R. J. Tetley, M. F. Staddon, D. Heller, A. Hoppe, S. Banerjee, and Y. Mao, Tissue fluidity promotes epithelial wound healing, Nature Physics **15**, 1195 (2019).

[2] E. Bitzek, P. Koskinen, F. Gähler, M. Moseler, and P. Gumbsch, Structural relaxation made simple, Physical Review Letters **97**, 170201 (2006).

[3] D. L. Barton, S. Henkes, C. J. Weijer, and R. Sknepnek, Active vertex model for cell-resolution description of epithelial tissue mechanics, PLoS Computational Biology **13**, e1005569 (2017).

[4] J. Delile, M. Herrmann, N. Peyriéras, and R. Doursat, A cell-based computational model of early embryogenesis coupling mechanical behaviour and gene regulation, Nature communications **8**, 13929 (2017).

[5] M. E. Newman and R. M. Ziff, Fast monte carlo algorithm for site or bond percolation, Physical Review E **64**, 016706 (2001).

[6] B. Aigouy, D. Umetsu, and S. Eaton, Segmentation and quantitative analysis of epithelial tissues, in *Drosophila: Methods and Protocols*, edited by C. Dahmann (Humana Press, New York, 2016) pp. 227–239.

[7] J. Kashef and C. M. Franz, Quantitative methods for analyzing cell–cell adhesion in development, Developmental Biology **401**, 165 (2015).

[8] M. Krieg, Y. Arboleda-Estudillo, P.-H. Puech, J. Käfer, F. Graner, D. Müller, and C.-P. Heisenberg, Tensile forces govern germ-layer organization in zebrafish, Nature Cell Biology **10**, 429 (2008).

[9] D. Biron, E. Alvarez-Lacalle, T. Tlusty, and E. Moses, Molecular model of the contractile ring, Physical Review Letters **95**, 098102 (2005).

[10] M. R. Stachowiak, C. Laplante, H. F. Chin, B. Guirao, E. Karatekin, T. D. Pollard, and B. O'Shaughnessy, Mechanism of cytokinetic contractile ring constriction in fission yeast, Developmental Cell **29**, 547 (2014).
